# MAFG-driven osteosarcoma cell progression is inhibited by a novel miRNA miR-4660

**DOI:** 10.1016/j.omtn.2021.03.006

**Published:** 2021-03-13

**Authors:** Hua-jian Shan, Lun-qing Zhu, Chen Yao, Zhi-qing Zhang, Yuan-yuan Liu, Qin Jiang, Xiao-zhong Zhou, Xiao-dong Wang, Cong Cao

**Affiliations:** 1 Department of Orthopedics, The Second Affiliated Hospital of Soochow University, Suzhou 215003, China; 2 Department of Pediatric Orthopedics, The Children’s Hospital of Soochow University, Suzhou 215100, China; 3 Department of Orthopedics, Affiliated Hospital of Nanjing University of Chinese Medicine, Jiangsu Province Hospital of TCM, Nanjing, China; 4 Jiangsu Key Laboratory of Neuropsychiatric Diseases and Institute of Neuroscience, Soochow University, Suzhou 215123, China; 5 Department of Radiotherapy and Oncology, Kunshan First People’s Hospital Affiliated to Jiangsu University, Kunshan, China; 6 The Affiliated Eye Hospital, Nanjing Medical University, Nanjing 210029, China; 7 The Affiliated Suzhou Hospital of Nanjing Medical University, Suzhou Municipal Hospital, Suzhou, China

**Keywords:** osteosarcoma, MAFG, miRNA-4660, Nrf2 signaling, molecularly targeted therapy

## Abstract

Osteosarcoma (OS) is the most common primary bone malignancy in the adolescent population. MAFG (v-maf avian musculoaponeurotic fibrosarcoma oncogene homolog G) forms a heterodimer with Nrf2 (NF-E2-related factor 2), binding to antioxidant response element (ARE), which is required for Nrf2 signaling activation. We found that *MAFG* mRNA and protein expression is significantly elevated in human OS tissues as well as in established and primary human OS cells. In human OS cells, MAGF silencing or knockout (KO) largely inhibited OS cell growth, proliferation, and migration, simultaneously inducing oxidative injury and apoptosis activation. Conversely, ectopic overexpression of MAFG augmented OS cell progression *in vitro*. MicroRNA-4660 (miR-4660) directly binds the 3′ untranslated region (UTR) of *MAFG* mRNA in the cytoplasm of OS cells. MAFG 3′ UTR luciferase activity and expression as well as OS cell growth were largely inhibited with forced miR-4660 overexpression but augmented with miR-4660 inhibition. *In vivo*, MAGF short hairpin RNA (shRNA) or forced overexpression of miR-4660 inhibited subcutaneous OS xenograft growth in severe combined immunodeficient mice. Furthermore, MAFG silencing or miR-4660 overexpression inhibited OS xenograft *in situ* growth in proximal tibia of the nude mice. In summary, MAFG overexpression-driven OS cell progression is inhibited by miR-4660. The miR-4660-MAFG axis could be novel therapeutic target for human OS.

## Graphical abstract

**Figure:**
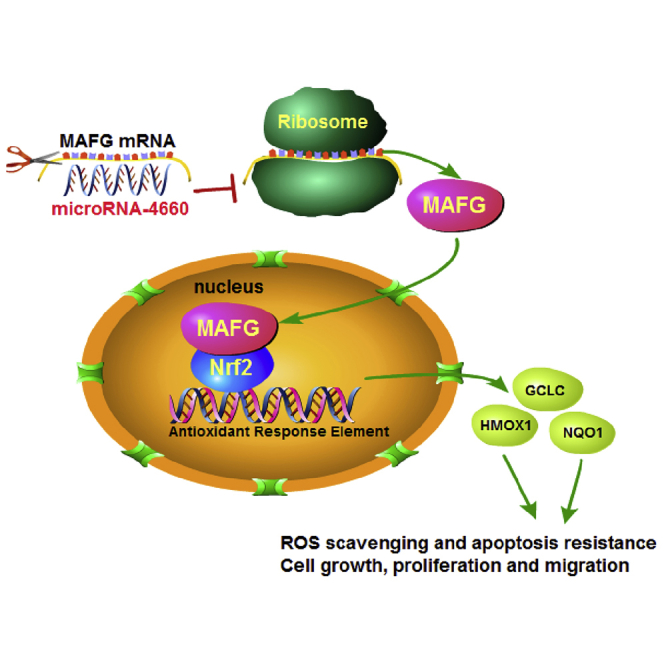

## Teaser

MAFG overexpression is involved in osteosarcoma tumorigenesis and progression. A novel microRNA, miR-4660, selectively silenced MAFG to inhibit osteosarcoma cell progression *in vitro* and *in vivo*. Targeting this miR-4660-MAFG axis could be a promising therapeutic strategy against osteosarcoma.

## Introduction

Osteosarcoma (OS) is the most common histological form of primary bone cancer in adolescents and children.^1^ It ranks the eighth-most-common form of childhood cancer, comprising around 2.4% of all cancers in pediatric patients and around one-fifth of all primary bone malignancies.^2^^,^^3^ Advanced OS is characterized by high malignancy grade, dismal prognosis, early local recurrence, and distant metastasis.^2^^,^^3^ OS patients can develop micro-metastases in the lung with dismally low 5-year survival rates.^4^ The standard treatment options for OS patients include surgery combined with chemotherapy.^5^^,^^6^ The large cooperative group studies have implied that there has been little to no further improvement of overall survival for OS patients in the past years.^6^ To improve clinical outcome, there is an urgent need to explore the pathological mechanisms of OS tumorigenesis and progression.^7^^,^^8^ Recent molecular genetic studies of OS have changed our view on the disease.^5^^,^^9^ The molecularly targeted therapies are the current research focus of OS.^2^^,^^5^^,^^9^

MAFG (v-maf avian musculoaponeurotic fibrosarcoma oncogene homolog G) is a small MAF protein belonging to the basic leucine zipper (bZIP) family of transcription factors.^10^ The MAFG bZIP structure has a basic DNA binding region and a leucine zipper structure^11^ but no canonical transcriptional activation domain.^11^ *MAFG* is broadly expressed in all human tissues but is relatively abundant in lung, lymph node, skeletal muscle, and thyroid tissues.^12^ MAFG knockout (KO) mice presented with a mild neuronal phenotype and mild thrombocytopenia.^13^

MAFG forms heterodimers with Nrf2 (NF-E2-related factor 2) protein, the latter being an essential regulator of antioxidant genes and detoxification enzymes.^14^^,^^15^ MAFG-Nrf2 heterodimers bind and activate the antioxidant response element (ARE) in the promoter regions of many genes involved in antioxidant defense.^16^ These genes including *heme oxygenase 1* (*HMOX-1*), *NAD(P)H quinone oxidoreductase 1* (*NQO1*), *glutamate cysteine ligase catalytic subunit* (*GCLC*), and many others.^14^^,^^15^ MAFG KO mouse embryonic fibroblasts (MEFs) failed to induce the Nrf2 signaling cascade in response to stress.^10^ In this study, we examine the expression and potential functions of MAFG in human OS. We show that MAFG overexpression is important for OS cell growth *in vitro* and *in vivo*.

MicroRNAs (miRNAs) are short non-coding RNAs with a length of ∼22 nucleotides. miRNAs participate in posttranscriptional regulation of gene expression,^17^ important for regulating almost all cellular and physiological behaviors, including cell differentiation, proliferation, and survival.^18^, ^19^, ^20^ miRNAs bind complementary target mRNAs, resulting in mRNA translational inhibition and/or degradation.^21^ miRNA dysregulation is detected in human OS,^22^, ^23^, ^24^ associated with OS tumorigenesis, progression, and therapy resistance.^25^^,^^26^ Here we identify a MAFG-targeting miRNA: microRNA-4660 (miR-4660). We show that miR-4660 specifically directly binds and silences MAFG to inhibit OS cell progression.

## Results

### MAFG overexpression in human OS

**Figure 1:**
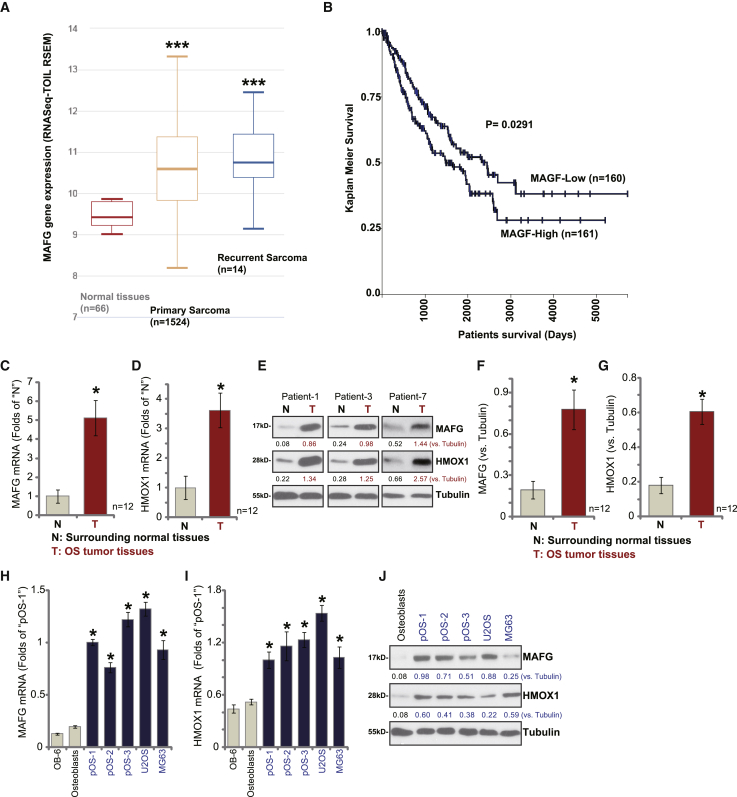
MAFG is upregulated in human OS tissues and cells The TARGET Pan-Cancer (PANCAN) database shows *MAFG* expression (RNA-seq-TOIL RSEM) in 1,604 cases of children’s sarcoma tissues, 14 cases of recurrent sarcoma samples, and 66 cases of normal adjacent tissues (A). Kaplan-Meier survival analyses of MAFG-low (n = 160) and MAFG-high (n = 161) children’s sarcoma patients (B). Expression of *MAFG*-*HMOX1* mRNA and protein in OS tumor tissues (T) and the surrounding normal tissues (N) of 12 primary human OS patients was shown, with results quantified (C–G). Expression of *MAFG*-*HMOX1* mRNA and protein in established OS cell lines (U2OS and MG63) and primary human OS cells, as well as in OB-6 human osteoblastic cells (OB-6) and primary human osteoblasts (osteoblasts) is shown, with results quantified. (H–J). Data were presented as mean ± standard deviation (SD). ∗∗∗p < 0.01 versus normal tissues (A). ∗p < 0.05 versus N tissues/osteoblasts (C–I).

OS comprises almost 60% of all the common histological subtypes of bone sarcoma.^3^ To examine *MAFG* expression in OS, we first consulted the TARGET Pan-Cancer (PANCAN) database to examine RNA sequencing (RNA-seq) data in children’s sarcoma tissues and normal adjacent tissues via a UCSC Xena project. As shown, in children’s sarcoma tissue (n = 1,524) *MAFG* mRNA expression is significantly upregulated (p < 0.05 versus normal tissues; n = 66) (Figure 1A). Furthermore, *MAFG* mRNA upregulation is detected in 14 cases of recurrent children’s sarcoma samples (Figure 1A). Importantly, the average survival of patients with *MAFG*-high sarcoma (RNA-seq ≥ 4.210) is lower than those with *MAFG*-low (MAFG RNA-seq < 4.210) sarcoma (p = 0.029; Figure 1B). These results show that *MAFG* is upregulated in children’s sarcoma tissues and is associated with poor overall survival.

To confirm the significance of the bioinformatics observations, we examined *MAFG* expression in human OS tissues. OS tumor tissues (T) and surrounding normal bone tissues (N), derived from a total of 12 primary OS patients, were analyzed. By quantitative real-time PCR, *MAFG* mRNA expression in OS tissues was 5.2-fold greater than in normal tissues (p < 0.05; Figure 1C). The mRNA expression of *heme oxygenase-1* (*HMOX1*), a key Nrf2 response gene,^27^^,^^28^ was also upregulated in OS tissues (p < 0.05 versus N tissues; Figure 1D). Western blotting studies were performed to test protein expression and showed that MAFG and HMOX1 protein expression was elevated in human OS tissues (Figure 1E). Quantification analyses integrating blotting data from 12 sets of human tissues confirmed that MAFG and HMOX1 protein upregulation was significant (p < 0.05 versus N tissues; Figures 1F and 1G).

We also examined MAFG expression in established OS cell lines (U2OS and MG63) and primary human OS cells. The primary OS cells were derived from three primary OS patients, namely pOS-1, pOS-2, and pOS-3 (see Materials and methods). As shown *MAFG-HMOX1* mRNA expression is relatively low in OB-6 human osteoblastic cells and primary human osteoblasts (Figures 1H and 1I). In the established and primary human OS cells, *MAFG-HMOX1* mRNA upregulation was detected (Figures 1H and 1I). Western blotting results (Figure 1J) confirmed MAFG-HMOX1 protein overexpression in established (U2OS and MG63) and primary OS cells, whereas low expression is detected in primary human osteoblasts. Together, these results confirmed MAFG overexpression in human OS.

### MAFG shRNA or KO inhibits OS cell viability, growth, proliferation, and migration

A set of three different lentiviral short hairpin RNAs (shRNAs), targeting non-overlapping sequences of *MAFG* (namely, sh-MAFG-Seq1/2/3; Table 1), were transduced to pOS-1 cells. Stable cells were established through puromycin selection. As shown, all applied shRNAs led to dramatic downregulation of *MAFG* mRNA and protein in pOS-1 cells (Figures S1A and S1B). Among the three shRNAs, sh-MAFG-Seq3 produced most significant MAFG knockdown efficiency. This shRNA, namely sh-MAFG, was selected for further experiments. Alternatively, the CRISPR-Cas9 gene editing method was applied to obtain complete MAFG KO. The pOS-1 cells were transduced with a lenti-CRISPR-Cas9-MAFG KO construct. Following MAFG-KO screening and puromycin selection, the monoclonal stable MAFG KO pOS-1 cells were established (ko-MAFG cells). Control cells were transduced with the scramble control shRNA plus the lenti-CRISPR-Cas9 empty vector (shC+Cas9 cells).

**Table 1:** Quantitative real-time PCR primers

Gene name	Forward primer (5′–3′)	Reverse primer (5′–3′)
*MAFG*	GTGGACAGGAAGCAGCTCA	TATTGGGGGTCGTCATAACC
*HMOX1*	GCTACCTGGGTGACCTGTCT	GGGCAGAATCTTGCACTTTG
*NRF2*	TGAGCATGCTTCCCATGAT	CTTCTCTAGCCGCTCTGTGG
*U6 RNA*	CTCGCTTCGGCAGCACATATACT	ACGCTTCACGAATTTGCGTGTC
*miR-4660*	TGCAGCTCTGGTGGAAAATG	GAACATGTCTGCGTATCTC

MAFG shRNA sequences: sh-MAFG-Seq1: CCGGCCTGTCCAAGGAGGAGATCGTCTCGAGACGATCTCCTCCTTGGACAGGTTTTT; sh-MAFG-Seq2: CCGGTGTTTCCTTCTCTTCTCTTTCCTCGAGGAAAGAGAAGAGAAGGAAACATTTTTTG; sh-MAFG-Seq3: CCGGGAGATCGTCCAGCTGAAGCAGCTCGAGCTGCTTCAGCTGGACGATCTCTTTTTTG; MAFG expression sequence: TTTTGTAATACGACTCACTATAGGGCGGCCGGGAATTCGTCGACTGGATCCGGTACCGAGGAGATCTGCCGCCGCGATCGCCATGACGACCCCCAATAAAGGAAACAAGGCCTTGAAGGTGAAGCGGGAGCCGGGTGAGAATGGCACCAGCCTGACGGATGAGGAGCTGGTGACCATGTCGGTGCGGGAGCTGAACCAGCACCTGCGGGGCCTGTCCAAGGAGGAGATCGTCCAGCTGAAGCAGCGCCGGCGCACGCTCAAGAACCGCGGCTACGCTGCCAGCTGCCGCGTGAAGCGGGTGACGCAGAAGGAGGAGCTGGAGAAGCAGAAGGCGGAGCTGCAGCAGGAGGTGGAGAAGCTGGCCTCAGAGAACGCCAGCATGAAGCTGGAGCTCGACGCGCTGCGCTCCAAGTACGAGGCGCTGCAGACCTTCGCCCGGACGGTGGCCCGCAGCCCCGTGGCGCCAGCCCGGGGCCCCCTTGCCGCCGGCCTGGGGCCCCTCGTCCCAGGCAAGGTGGCCGCCACCAGCGTCATCACAATAGTAAAGTCCAAGACGGATGCCCGATCGACGCGTACGCGGCCGCTCGAGCAGAAACTCATCTCAGAAGAGGATCTGGCAGCAAATGATATCCTGGATTACAAGGATGACGACGATAAGGTTTAA. Pre-miR-4660 expression sequence: GCAACTGTGCTCAACCTTGTCTGCGTACTGGAGTCATCAAGGAAGCTGCGACCAGCGCAGACGCTGGGCTGTCATCTCTGGAGGCACTGATTTGCTTCATCGATCTGAGAGCAGCTTGGGCATAGGGATTTGTTTTTTTTAAGACTGAAAATCACTGCATTAGGGAAGCAGTGATGAGAATTCAATAATTCGAATTACATGTCAGCACTGATGTGAGGGTTCAAGATAGGAATTTCCCTCCTGGCACATTACTCAATTGATAGTTTAAAAAACGTCCTTAAGAAGAAGAAAGACAGCCAAACTCCTTCTGCAGCTCTGGTGGAAAATGGAGAAGACTTTTCCTTTCCTCCATCTCCCCCAGGGCCTGGTGGAGTGAGGCGTTGCCCAGCTATAAACTGTGGTCACTCTTCTGGTGGGGCCAGCAGATAACAGCTGATGTGTGAAGGAGGCAGGGAAGGACATGGGGAAAAGGTCCAGTGGAAATCAAAGCCCATTGTCATAATTCTGTCCTTTGTTTTGAGACAGGATCTTGCTCCGTTGTCTAGGCTGGAGTGCAGTGGTGCAATCATAGTTGACTGCAGCCTCAATCTCCCTAGCTCAGGTGATCCTCCCACCTCAGCTTCCTGAGTAGCTGGGACCACAGGTACGTGCCCCTGGCTAATTATTATTATTTT

**Figure 2:**
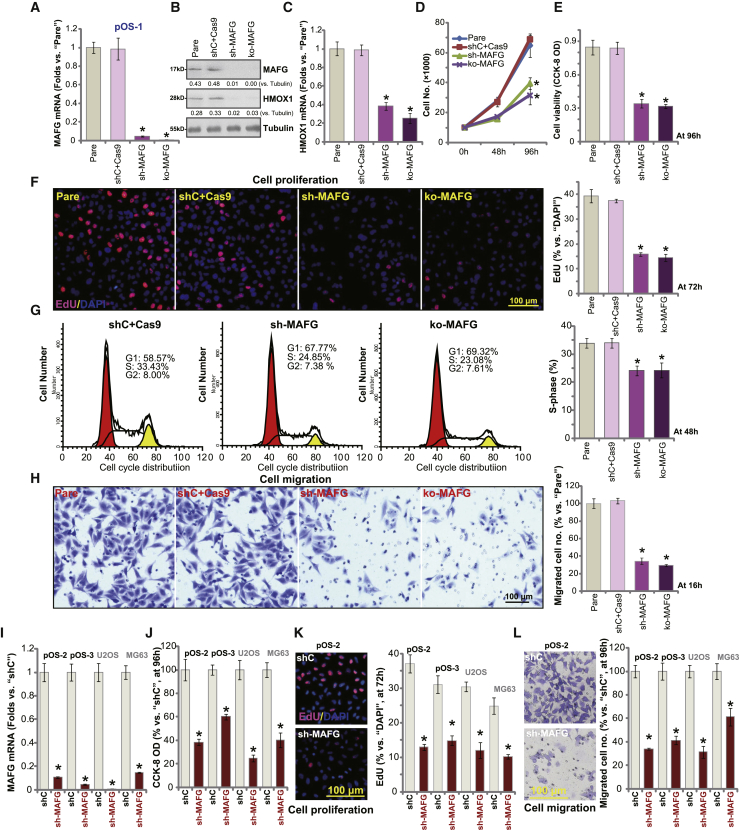
MAFG shRNA or KO inhibits OS cell viability, growth, proliferation, and migration Stable primary human OS cells (pOS-1/pOS-2/pOS-3, derived from OS patients) or the established OS cell lines (U2OS and MG63), bearing MAFG shRNA (sh-MAFG), the lenti-CRISPR-Cas9-MAFG-KO construct (ko-MAFG), scramble control shRNA plus CRISPR-Cas9 empty vector (shC+Cas9), or scramble control shRNA (shC), were established and expression of listed genes tested by quantitative real-time PCR and western blotting assays (A–C and I). Cells were cultured for applied time periods, cell growth (D), viability (CCK-8 assays, E and J) and proliferation (nuclear EdU incorporation, F and K), as well as cell cycle progression (G) and cell migration (H and L) (Transwell assays) were tested by the assays mentioned in the text, with results quantified. For EdU staining assays, five random views with a total of 1,000 cell nuclei from each treatment were included to calculate the average EdU/DAPI ratio (same for all figures). For Transwell/Matrigel Transwell assays, five random views were included to calculate the average number of migrated/invaded cells in each condition (same for all figures). For all the functional assays, the exact same number of viable cells of different genetic treatments were seeded initially to each well or each dish (at 0 h/day 0, same for all figures). Pare, parental control cells (same for all figures). Expression of listed proteins was quantified and normalized to the loading control (B). Data are presented as mean ± standard deviation (SD, n = 5). ∗p < 0.05 versus shC+Cas9 cells/shC cells. Experiments in this figure were repeated five times, with similar results obtained.

The quantitative real-time PCR assay results (Figure 2A) demonstrated that *MAFG* mRNA expression decreased by 95% in sh-MAFG (by sh-MAFG-Seq3) cells and ko-MAFG cells (p < 0.05 versus shC+Cas9 cells). Furthermore, MAFG protein expression was robustly downregulated (Figure 2B). MAFG shRNA or KO led to downregulation of *HMOX1* mRNA (Figure 2C) and protein (Figure 2B) in pOS-1 cells, indicating Nrf2 signaling inhibition. Two other MAFG shRNAs, sh-MAFG-Seq1/2, also downregulated *HMOX1* mRNA in pOS-1 cells (Figure S1C). To further confirm Nrf2 inhibition, we showed that the relative ARE-reporter luciferase activity in pOS-1 cells was significantly downregulated by MAFG shRNA or KO (Figure S2A). Additionally, mRNA expressions of two other Nrf2-dependent genes, *NQO1* and *GCLC*, were decreased in MAFG-silenced/-KO pOS-1 cells (Figures S2B and S2C).

The cell growth curve results (Figure 2D) demonstrated that the growth of sh-MAFG cells and ko-MAFG cells was much slower than shC+Cas9 control cells (p < 0.05). Testing cell viability using a Cell Counting Kit-8 (CCK-8) assay, we found that MAFG silencing or KO resulted in a 60%–70% viability reduction in pOS-1 cells (Figure 2E; p < 0.05 versus shC+Cas9 cells). Nuclear 5-ethynyl-20-deoxyuridine (EdU) incorporation (% versus 4',6-diamidino-2-phenylindole [DAPI]) was also decreased in MAFG-silenced/-KO pOS-1 cells (Figure 2F; p < 0.05 versus shC+Cas9 cells), suggesting proliferation inhibition. Notably, in pOS-1 cells the two other MAFG shRNAs (sh-MAFG-Seq1/2) also led to viability (CCK-8 optical density [OD]) reduction (Figure S1D) and proliferation (EdU-positive nuclei ratio) inhibition (Figure S1E).

Examining cell cycle progression, using the propidium iodide-fluorescence-activated cell sorting (PI-FACS) assay, confirmed that MAFG shRNA or KO induced G1-S arrest in pOS-1 cells, as both increased the number of G1-phase cells but decreased S- and G2-phase cells (Figure 2G; p < 0.05 versus shC+Cas9 cells). Furthermore, pOS-1 cell migration, tested by Transwell assay (Figure 2H), was robustly inhibited by MAFG shRNA or KO (p < 0.05 versus shC+Cas9 cells).

The potential pro-cancerous function of MAFG was tested in additional human OS cells. In primary human OS cells derived from two other OS patients, pOS-2 and pOS-3, as well as in established OS cell lines (U2OS and MG63), the application of the MAFG-shRNA lentivirus led to an 80%–90% reduction of *MAFG* mRNA (Figure 2I; p < 0.05 versus shC cells). Functional studies demonstrated that MAFG shRNA in OS cells potently inhibited cell viability (CCK-8 OD; Figure 2J; p < 0.05 versus shC cells), cell proliferation (nuclear EdU ratio; Figure 2K; p < 0.05 versus shC cells), and migration (Figure 2L; p < 0.05 versus shC cells). Collectively, these results show that MAFG silencing or KO inhibited cell viability, growth, proliferation, and migration in established and primary human OS cells.

### MAFG shRNA or KO induces oxidative injury and apoptosis in OS cells

**Figure 3:**
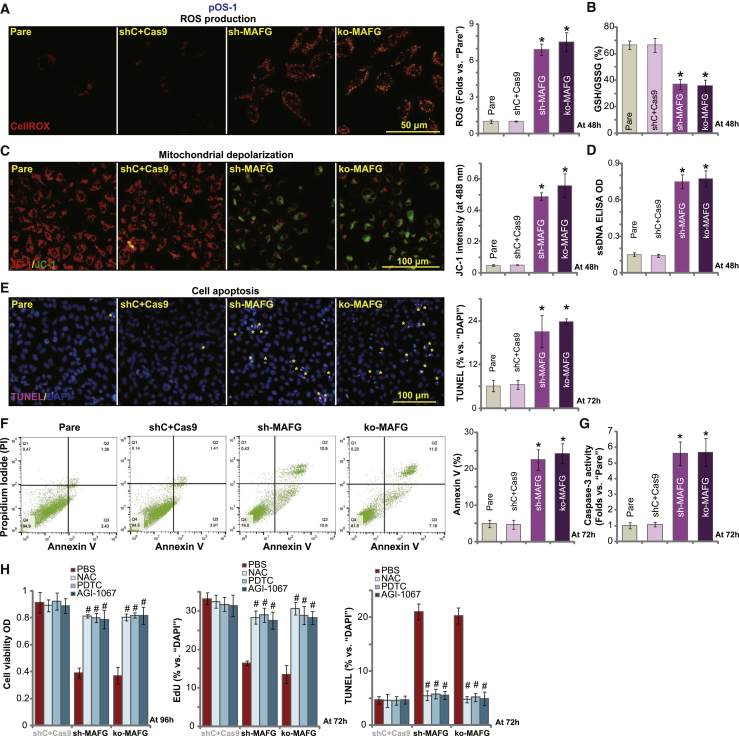
MAFG shRNA or KO induces oxidative injury and apoptosis in OS cells Stable pOS-1 cells, bearing MAFG shRNA (sh-MAFG), the lenti-CRISPR-Cas9-MAFG-KO construct (ko-MAFG), and scramble control shRNA plus CRISPR-Cas9 empty vector (shC+Cas9), were cultured for applied time periods; ROS levels were tested by measuring CellROX intensity (A) and the GSH/GSSG ratio (B). Mitochondrial depolarization was tested by the fluorescence dye JC-1 (C), with single-strand DNA (ssDNA) contents measured by an ELISA kit (D). Cell apoptosis was examined and quantified by nuclear TUNEL staining (E), Annexin V FACS (F), and Caspase-3 activity (G) assays. pOS-1 cells with shC+Cas9, sh-MAFG, or ko-MAFG were treated with NAC (50 μM), PDTC (40 μM), or AGI-1067 (10 μM) and cultured for applied time periods; cell viability (CCK-8 OD), proliferation (nuclear EdU incorporation), and apoptosis (TUNEL staining) were tested, with results quantified (H). Data were presented as mean ± standard deviation (SD, n = 5). ∗p < 0.05 versus shC+Cas9 cells. ^#^p < 0.05 versus PBS treatment (H). Experiments in this figure were repeated five times with similar results obtained.

As MAFG silencing or KO resulted in inhibition of the Nrf2 cascade in OS cells (Figure 2), we therefore tested whether this would provoke reactive oxygen species (ROS) production and oxidative injury. The intracellular ROS contents, reflected by the CellROX fluorescein intensity,^29^ were significantly increased in sh-MAFG pOS-1 cells and ko-MAFG pOS-1 cells (Figure 3A; p < 0.05 versus shC+Cas9 cells). Furthermore, MAFG shRNA or KO reduced the reduced glutathione (GSH)/oxidized glutathione disulfide (GSSG) ratio (p < 0.05 versus shC+Cas9 cells), further indicating oxidative injury (Figure 3B). Additionally, JC-1 green monomer accumulation, an indicator of mitochondrial depolarization, was detected in MAFG-silenced/MAFG-KO pOS-1 cells (Figure 3C), which was accompanied by single-strand DNA (ssDNA) accumulation (indicating DNA damage; Figure 3D). Therefore, MAFG shRNA or KO induced profound oxidative injury in pOS-1 cells, causing ROS production, GSH/GSSG ratio reduction, mitochondrial depolarization, and DNA damage.

In OS cells, proliferation arrest and/or oxidative injury is anticipated to result in cell apoptosis. Testing the potential effect of MAFG depletion on cell apoptosis, we found that the ratio of terminal deoxynucleotidyl transferase (TdT)-mediated dUTP nick end labeling (TUNEL)-positive nuclei was significantly increased in pOS-1 cells-bearing the MAFG-shRNA or MAGF-KO construct (Figure 3E; p < 0.05 versus shC+Cas9 cells). Annexin V FACS assay results (Figure 3F) further confirmed that MAFG silencing or KO caused apoptosis activation. Significant caspase-3 activation was also detected in pOS-1 cells with MAFG silencing or KO (Figure 3G). The two other MAFG shRNAs, sh-MAFG-Seq1/2, induced ROS production (CellROX intensity increase; Figure S1F), mitochondrial depolarization (JC-1 green monomer intensity increase; Figure S1G), and apoptosis activation (nuclear TUNEL ratio increase; Figure S1H) in pOS-1 cells. These results confirm that MAFG silencing/depletion induces robust apoptosis activation in primary OS cells.

To test whether oxidative stress is the primary cause of cell apoptosis in MAFG-depleted cells, we applied several known antioxidants, including N-acetylcysteine (NAC), pyrrolidine dithiocarbamate (PDTC),^30^^,^^31^ and AGI-1067.^32^^,^^33^ As shown, in pOS-1 cells MAFG shRNA- and MAFG KO-induced viability (CCK-8 OD) reduction, proliferation inhibition (nuclear EdU ratio reduction), and cell apoptosis (increased ratio in TUNEL-positive nuclei) were largely attenuated by the ROS scavengers (Figure 3H). These results indicate MAFG silencing/KO resulted in ROS accumulation to induce OS cell apoptosis.

In other primary (pOS-2 and pOS-3) and established OS cells (U2OS and MG63), MAFG silencing by targeted lentiviral shRNA (sh-MAFG-Seq3/2) similarly induced robust ROS production (CellROX intensity increase; Figure S1I), mitochondrial depolarization (JC-1 green monomers accumulation; Figure S1J), and cell apoptosis (TUNEL-positive nuclei ratio increase; Figure S1K). These results together suggest that MAFG shRNA or KO induces oxidative injury and apoptosis in human OS cells.

### Ectopic MAFG overexpression further promotes OS cell progression *in vitro*

**Figure 4:**
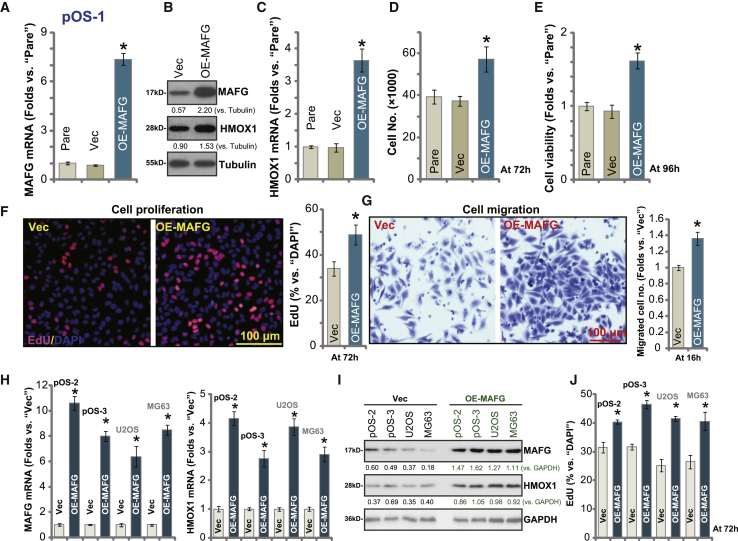
Ectopic MAFG overexpression further promotes OS cell progression *in vitro* Stable primary human OS cells (pOS-1/pOS-2/pOS-3, derived from different OS patients) or the established OS cell lines (U2OS and MG63), bearing the MAFG-expressing GV369 vector (OE-MAFG) or the empty vector (Vec), were established and cultured; expression of listed genes was tested by western blotting and quantitative real-time PCR assays (A–C, H, and I). Cells were further cultured for applied time periods; cell growth (D), viability (E), proliferation (F and J), and migration (G) were tested by the assays mentioned in the text. Data were presented as mean ± standard deviation (SD, n = 5). ∗p < 0.05 versus Vec cells. Experiments in this figure were repeated five times with similar results obtained.

Based on the results, we hypothesized that MAFG overexpression would promote OS cell progression *in vitro*. A MAFG-expressing GV369 vector was transduced into pOS-1 cells, and stable cells were established (OE-MAFG cells). Quantitative real-time PCR assay results (Figure 4A) confirmed that *MAFG* mRNA levels were increased over seven-fold in the OE-MAFG cells, and MAFG protein expression was significantly elevated (Figure 4B). Further, *HMOX1* mRNA and protein expression was increased (Figures 4B and 4C). Overexpression of MAFG augmented ARE-reporter luciferase activity (Figure S2D), indicating Nrf2 cascade activation, which was further confirmed by increased mRNA expression of *NQO1* (Figure S2E) and GCLC (Figure S2F).

Cell growth results (Figure 4D) showed that OE-MAFG cells grew significantly faster than control cells. In pOS-1 cells, ectopic overexpression of MAFG augmented cell viability (CCK-8 OD; Figure 4E) and proliferation (EdU ratio; Figure 4F). Furthermore, cell migration (Figure 4G) was enhanced in MAFG-overexpressed pOS-1 cells.

Similarly, in other primary OS cells (pOS-2/pOS-3) and established OS cell lines (U2OS and MG63), transfection of the MAFG-expressing GV369 vector resulted in upregulation of *MAFG* mRNA (Figure 4H). Consequently, *HMOX1* mRNA levels were significantly increased (Figure 4H). Increased MAFG and HMOX1 protein levels were detected in the OS cells with MAFG-expressing vector (Figure 4I). EdU incorporation assay results (Figure 4J) demonstrated that ectopic overexpression of MAFG promoted cell proliferation in OS cells. Thus, ectopic overexpression of MAFG promotes human OS cell progression *in vitro*.

### miR-4660 directly binds and silences *MAFG* in OS cells

**Figure 5:**
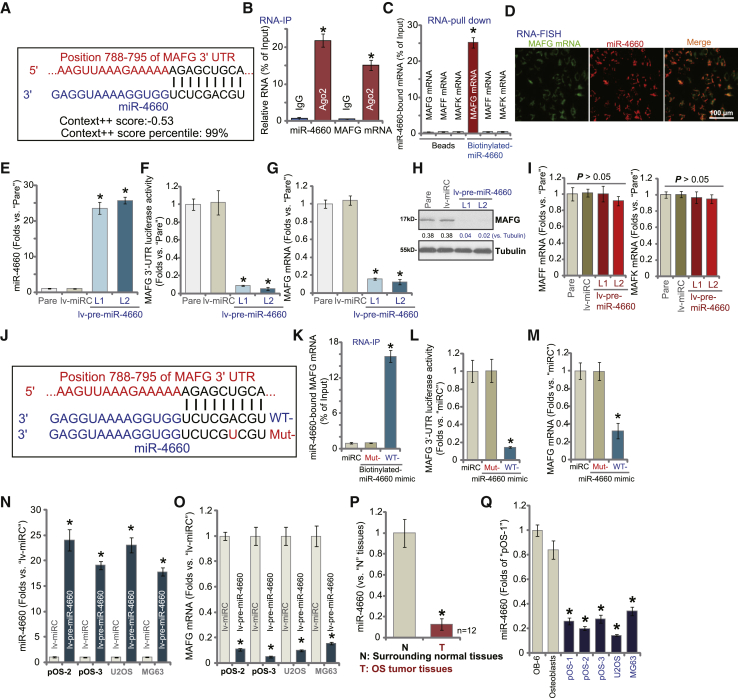
miR-4660 directly binds and silences *MAFG* in OS cells miRNA-4660 putatively targets the 3′ UTR (untranslated region) of *MAFG mRNA* (at position of 788–795) (A). miRNA-4660 (fluorescence-tagged) locates in the cytoplasm of pOS-1 cells (B). RNA immunoprecipitation (RNA-IP) experiment results demonstrate that the Ago2 protein immunoprecipitated with *MAFG* mRNA and miR-4660 in pOS-1 cells (B). RNA-pull-down assay results display that biotinylated-miR-4660 associated with *MAFG* mRNA (but not *MAFF* mRNA and *MAFK* mRNA) in pOS-1 cells (C). Fluorescence *in situ* hybridization (FISH) results show miR-4660 (in red) and *MAFG* mRNA (in green) cellular distribution and their co-localization in pOS-1 cells (D).The pOS-1 cells were transduced with lentiviral pre-microRNA-4660 (lv-pre-miR-4660). With selection by puromycin, two stable cell lines were established: lv-pre-miR-4660-L1/lv-pre-miR-4660-L2. Control cells were transduced with the lentiviral nonsense microRNA (lv-miRC) construct. Expression of mature miR-4660 and listed mRNAs was tested by quantitative real-time PCR (E, G, and I). The MAFG 3′ UTR luciferase reporter activity was tested (F), with expression of listed proteins tested by western blotting (H). pOS-1 cells were transfected with 500 nM of nonsense microRNA control (miRC), the wild-type (WT), or the mutant miR-4660 mimic (sequences listed in J). miR-4660-MAFG mRNA binding was tested by RNA pull-down (K). The MAFG 3′ UTR luciferase reporter activity (L), as well as its mRNA expression (M) were tested. The applied OS cells, including pOS-2, pOS-3, U2OS, and MG63, were infected with lv-pre-miR-4660 or lv-miRC for 72 h; expression of miR-4660 (N) and *MAFG* mRNA (O) is shown. Expression of miR-4660 in OS tumor tissues (T) and the surrounding normal tissues (N) of 12 primary human OS patients is shown, with results quantified (P). Expression of miR-4660 in established OS cell lines (U2OS and MG63) and primary human OS cells, as well as in OB-6 human osteoblastic cells (OB-6) and primary human osteoblasts (osteoblasts) is shown, with results quantified (Q). Data are presented as mean ± standard deviation (SD). ∗p < 0.05 versus IgG or bead control (B and C). ∗p < 0.05 versus lv-miRC/miRC/lv-anta-C cells. ∗p < 0.05 versus N tissues/osteoblasts (P and Q). Experiments in this figure were repeated five times with similar results obtained.

To identify *MAFG*-targeting miRNAs that bind its 3′ UTR, we searched the microRNA database, TargetScan (V7.2).^34^ Promising *MAFG*-targeting miRNAs were further verified through multiple microRNA databases: miRbase (v21.0) and miRDB. Three candidate miRNAs with the context score percentage over 99% and the context++ score less than −0.5 were retrieved, including miR-4660, miR-4730, and miR-1184. Each of the three miRNA mimics (500 nM, 48 h) were individually transfected to pOS-1 cells, of which only miR-4660 mimic resulted in significant MAFG mRNA downregulation (Figure S3A). As shown, microRNA-4660 (miR-4660) putatively targets the *MAFG* 3′ UTR (at position 788–795; Figure 5A). The miR-4660-*MAFG* 3′ UTR binding context score percentage is 99%, with the context^++^ score at −0.53 (TargetScan V7.2;^34^ Figure 5A). These results indicate a high percentage of direct binding between the two.^34^

By employing an RNA-immunoprecipitation (RNA-IP) assay, we demonstrated that endogenous *MAFG* mRNA and miR-4660 were pulled down by an anti-Ago2 antibody in pOS-1 cells (Figure 5B). The non-specific anti-immunoglobulin G (IgG) antibody failed to pull down *MAFG* mRNA and miR-4660 (Figure 5B). RNA pull-down assay results further showed that biotinylated-miR-4660 directly associated with *MAFG* mRNA, but not with two other MAF family genes, *MAFF* mRNA and *MAFK* mRNA (Figure 5C). In addition, fluorescence *in situ* hybridization (FISH) assay results demonstrated that miR-4660 (in red) and *MAFG* mRNA (in green) were co-localized mainly in the cytoplasm of pOS-1 cells (Figure 5D). These results suggested a high probability for the two to bind.^34^

Based on the above results, we hypothesized that ectopic miR-4660 overexpression would alter MAFG expression. A lentiviral construct encoding pre-miRNA-4600 (lv-pre-miR-4660) was transduced into pOS-1 cells, and two stable cell lines, lv-pre-miR-4660-L1 and lv-pre-miR-4660-L2, were established via puromycin selection. The quantitative real-time PCR results (Figure 5E) confirmed that the expression of mature miRNA-4660 increased over 20-fold in lv-pre-miR-4660 cells (p < 0.05 versus cells with miRNA control construct/lv-miRC). A dual-luciferase reporter assay demonstrated that MAFG 3′ UTR luciferase reporter activity was significantly decreased after the forced miR-4660 overexpression in pOS-1 cells (p < 0.05 versus lv-miRC cells) (Figure 5F). Consequently, *MAFG* mRNA (Figure 5G) and protein (Figure 5H) expression were dramatically downregulated in miRNA-4660-overexpressed cells. Expression of *MAFF* mRNA and *MAFK* mRNA was unchanged, however (Figure 5I). In lv-pre-miR-4660-expressing pOS-1 cells, the relative ARE-reporter luciferase activity (Figure S2G) as well as *NQO1* (Figure S2H) and *GCLC* (Figure S2I) mRNA expression were largely inhibited, confirming Nrf2 cascade inhibition.

The results demonstrate that miRNA-4660 can selectively target and silence *MAFG* mRNA in OS cells. To further support this hypothesis, pOS-1 cells were transfected with either wild-type (WT) or the mutant (Mut) miR-4660 mimic. The mutant miR-4660 mimic (Mut-miR-4660) contains nucleotide mutations at the miR-4660 binding site to MAFG 3′ UTR (see sequence in Figure 5J). RNA-IP experiment results confirmed that only WT-miR-4660 immunoprecipitated with *MAFG* mRNA, while the mutant failed (Figure 5K). Transfection the WT-miR-4660, but not the Mut-miR-4660, decreased MAFG 3′ UTR luciferase reporter activity (Figure 5L) as well as expression of *MAFG* mRNA (Figure 5M) in pOS-1 cells.

We next tested whether miR-4660 inhibition could increase MAFG expression. A lentiviral construct encoding the anti-sense of pre-miR-4660, or lv-antagomiR-4660, was transduced into pOS-1 cells, and stable cells were established. As compared to cells transduced with the lentiviral microRNA anti-sense control construct (lv-anta-C), mature miR-4660 levels decreased over 90% in lv-antagomiR-4660-expressing pOS-1 cells (Figure S3B). Importantly, miR-4660 inhibition resulted in an increased MAFG 3′ UTR luciferase reporter activity (Figure S3C), as well as upregulation of *MAFG* mRNA (Figure S3D). Therefore, forced miR-4660 inhibition resulted in MAFG upregulation, further corroborating that miR-4660 is a MAFG-targeting miRNA in OS cells. In other primary OS cells (pOS-2/pOS-3) and established cells (U2OS and MG63), ectopic miR-4660 overexpression, using lv-pre-miR-4660 (Figure 5N), led to significant *MAFG* mRNA reduction (Figure 5O).

We also analyzed expression of miR-4660 in the human OS tissues described in Figure 1. The quantitative real-time PCR assay results (Figure 5P) demonstrated that miR-4660 expression in OS tumor tissues (T) was significantly lower than that in the surrounding normal tissue (N). Furthermore, low miR-4660 expression was detected in primary OS cells (pOS-1, pOS-2, and pOS-3) and established OS cell lines (U2OS and MG63) (Figure 5Q), compared to expression in OB-6 osteoblastic cells and primary human osteoblasts (Figure 5Q). Therefore, in human OS tissues, low miR-4660 expression is correlated with MAFG elevation (see Figure 1).

### miR-4660 overexpression inhibits OS cell progression *in vitro*

**Figure 6:**
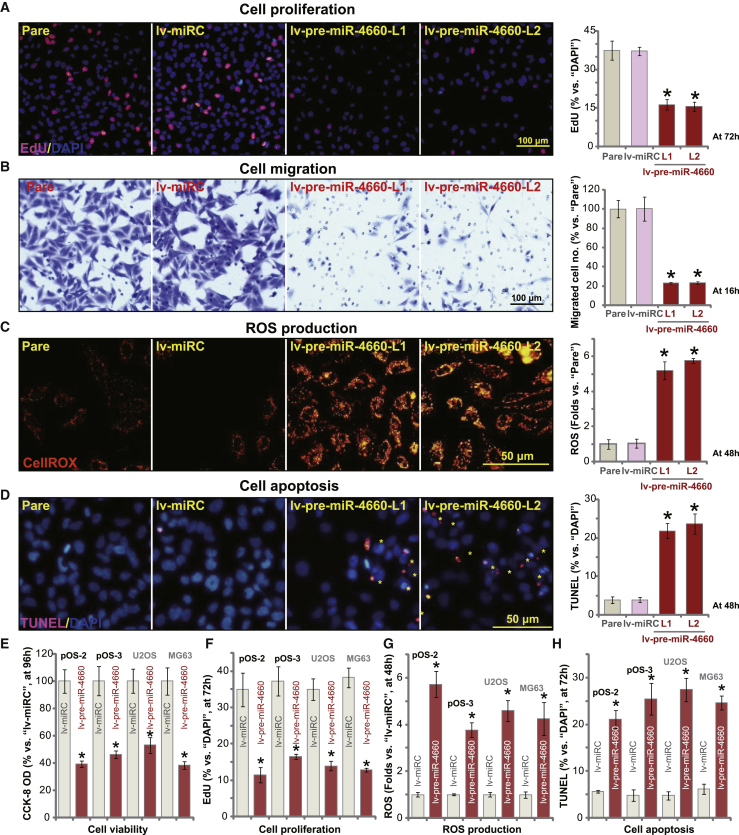
miR-4660 overexpression inhibits OS cell progression *in vitro* Stable primary human OS cells (pOS-1/pOS-2/pOS-3, derived from different OS patients) or the established OS cell lines (U2OS and MG63) were transduced with lentiviral pre-microRNA-4660 (lv-pre-miR-4660). Control cells were transfected with lentiviral nonsense microRNA (lv-miRC). With selection by puromycin, two stable cell lines were established. Cells were further cultured for applied time periods. Cell proliferation (by recording nuclear EdU incorporation, A and F) and migration (B) were tested by the assays mentioned in the text, with results quantified. Cellular ROS intensity and apoptosis were tested by CellROX staining (C and G) and TUNEL staining (D and H) assays, respectively. Cell viability was tested by CCK-8 assay (E). Data are presented as mean ± standard deviation (SD, n = 5). ∗p < 0.05 versus lv-miRC cells. Experiments in this figure were repeated five times with similar results obtained.

As miR-4660 can silence MAFG expression, we tested its effect on OS cell function. EdU incorporation assay results (Figure 6A) demonstrated that forced expression of miRNA-4660, using lv-pre-miR-4660 (see Figure 5), potently inhibited pOS-1 cell proliferation (recording EdU/DAPI ratio, p < 0.05 versus lv-miRC cells). Furthermore, *in vitro* cell migration was largely suppressed in pOS-1 cells bearing lv-pre-miR-4660 (Figure 6B; p < 0.05 versus lv-miRC cells). ROS production was enhanced, as indicated by increased CellROX fluorescence intensity detected in miR-4660-overexpressed pOS-1 cells (Figure 6C; p < 0.05 versus lv-miRC cells). Overexpression of miRNA-4660 also induced cell apoptosis, evidenced by increased ratio of TUNEL-positive nuclei (Figure 6D; p < 0.05 versus lv-miRC cells). Conversely, miR-4660 silencing by lv-antagomiR-4660 led to increased cell proliferation (Figure S3E) and migration (Figure S3F) in pOS-1 cells.

Treatment with the antioxidants, NAC and PDTC, largely inhibited miR-4660 overexpression (lv-pre-miR-4660-L1 and lv-pre-miR-4660-L2)-induced viability (CCK-8 OD) reduction (Figure S3G), proliferation inhibition (nuclear EdU staining assay; Figure S3H), and cell apoptosis (by recording nuclear TUNEL ratio; Figure S3I). These results support the model that miR-4660 overexpression leads to MAFG downregulation, ROS production, and oxidative injury, causing significant anti-OS cell activity.

In other primary OS cells (pOS-2 and pOS-3) and established cells (U2OS and MG63), forced overexpression of miR-4660 by lv-pre-miR-4660 (see Figure 5) inhibited cell viability (CCK-8 OD; Figure 6E) and proliferation (EdU ratio; Figure 6F). Furthermore, ROS production (CellROX intensity increase; Figure 6G) and apoptosis (TUNEL ratio increase; Figure 6H) were detected in lv-pre-miR-4660-expressing OS cells. Therefore, mimicking MAFG silencing-induced actions, miR-4660 overexpression inhibited pOS-1 cell proliferation and migration but provoked oxidative injury and cell apoptosis.

Significantly, in OE-MAFG pOS-1 cells, stable transfection of lv-pre-miR-4660 induced miR-4660 overexpression (Figure S3J), causing robust *MAFG* mRNA depletion (Figure S3K). Importantly, miR-4660 overexpression largely inhibited high basal cell proliferation (EdU positive nuclei ratio; Figure S3L) and migration (Figure S3M) in OE-MAFG pOS-1 cells.

### miR-4660-induced anti-OS cell activity is due to MAFG silencing

**Figure 7:**
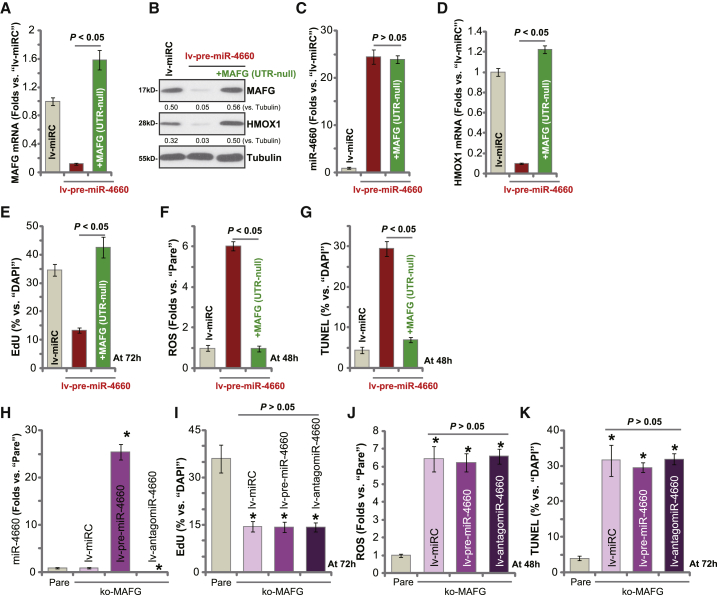
miR-4660-induced anti-OS cell activity is due to MAFG silencing Stable pOS-1 cells with the pre-miRNA-4600-expressing construct (lv-pre-miR-4660) were further transduced with or without a lentiviral 3′ UTR null MAFG expression vector: MAFG (UTR null). Control cells were with scramble control microRNA (lv-miRC). Expression of listed genes was tested by quantitative real-time PCR and western blotting assays (A–D). Cells were further cultured for applied time periods; cell proliferation, ROS production, and apoptosis were tested by EdU incorporation (E), CellROX intensity (F), and TUNEL staining (G) assays, respectively, with results quantified. Stable pOS-1 cells with the lenti-CRISPR-Cas9-MAFG-KO construct (ko-MAFG) were transduced with lv-pre-miR-4660, lv-antagomiR-4660, or lv-miRC; expression of mature miR-4660 is shown (H). Cells were further cultured for applied time periods; cell proliferation (I), ROS production (J), and apoptosis (K) were tested similarly. Pare, parental control cells. Data were presented as mean ± standard deviation (SD, n = 5). ∗p < 0.05 versus Pare cells (H–K). Experiments in this figure were repeated five times with similar results obtained.

To further confirm that miR-4660-induced anti-OS cell activity is due to MAFG silencing, a lentiviral 3′ UTR null MAFG expression vector, MAFG (UTR null), was constructed. As shown, the construct completely restored *MAFG* mRNA (Figure 7A) and protein (Figure 7B) expression in lv-pre-miR-4660-expressing pOS-1 cells but had no significant effect on miR-4660 expression (Figure 7C). Further, miR-4660 overexpression-induced *HMOX1* downregulation (both mRNA and protein) was reversed by the MAFG (UTR null) construct (Figures 7A and 7D). These results show that MAFG (UTR null) can restore MAFG-HMOX1 expression in miR-4660-overexpressed pOS-1 cells. Functional studies demonstrated that in pOS-1 cells, lv-pre-miR-4660-induced proliferation inhibition (EdU ratio reduction; Figure 7E), ROS production (CellROX intensity increase; Figure 7F), and cell apoptosis (TUNEL staining increase; Figure 7G) were reversed by MAFG re-expression.

Based on these results, altering miR-4660 expression should be ineffective in MAFG-depleted cells. The ko-MAFG cells (see Figures 2 and 3) were transduced with lv-pre-miR-4660 or lv-antagomiR-4660, both of which significantly altered mature miR-4660 expression (Figure 7H). MAFG-KO-induced proliferation inhibition (Figure 7I), ROS production (Figure 7J), and cell apoptosis (Figure 7K) were not affected by ectopic miR-4660 overexpression or inhibition (Figures 7I–7K). These results further supported that miR-4660 silenced MAFG to inhibit OS cell progression, and MAFG KO abolished its activity.

### MAFG silencing or miR-4660 overexpression inhibits growth of subcutaneous OS xenografts and *in situ* OS xenografts in mice

**Figure 8:**
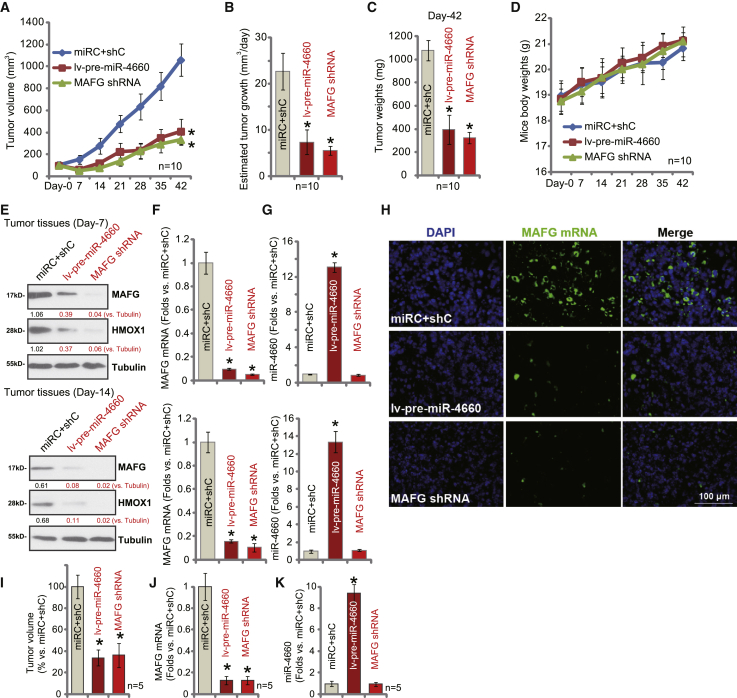
MAFG silencing or miR-4660 overexpression inhibits growth of subcutaneous OS xenografts and *in situ* OS xenografts in mice The SCID mice bearing pOS-1 xenografts were randomly assigned into three groups, intratumorally injected with MAFG shRNA lentivirus (MAFG shRNA), pre-miR-4660 lentivirus (lv-pre-miR-4660), or the control virus (miRC+shC); tumor volumes (A) or the mice body weights (D) were recorded every 7 days. Estimated daily tumor growth was calculated (B). At the end of the experiments (day 42), tumors were isolated and individually weighed (C). At day 7 and day 14, one tumor of each group was isolated, and total six tumors were achieved; expression of listed genes in tumor tissue lysates is shown (E–G). *In vivo* RNA-FISH in tumor slides further confirmed *MAFG* mRNA silencing in MAFG shRNA- and lv-pre-miR-4660-tumors (H). At 3 × 10^6^ cells per mouse, stable pOS-1 cells expressing MAFG shRNA lentivirus (MAFG shRNA), pre-miR-4660 lentivirus (lv-pre-miR-4660), or the control virus (miRC+shC) were injected into the proximal tibia of the nude mice. Twenty-four days after injection (day 24), *in situ* tumor volumes were recorded (I). Tumors were also isolated; expression of listed genes in tumor tissue lysates is shown (J and K). Data are presented as mean ± standard deviation (SD). ∗p < 0.05 versus miRC+shC tumors.

To study the potential role of the miR-4660-MAFG axis on OS cell growth *in vivo*, a xenograft mouse model was applied. The pOS-1 cells were subcutaneously (s.c.) injected into the flanks of severe combined immunodeficient (SCID) mice. Within 3 weeks, OS xenografts were established, with each tumor close to 100 mm^3^. Mice were then randomly assigned into three groups (day 0), with intratumoral injection of MAFG shRNA lentivirus (MAFG shRNA; see Figure 2), pre-miR-4660 lentivirus (lv-pre-miR-4660; see Figure 5), or the control virus (miRC+shC). The tumor growth curve results confirmed that tumors bearing MAFG shRNA or lv-pre-miR-4660 grew significantly slower than the control tumors (Figure 8A). The estimated daily tumor growth, calculated by the formulation (tumor volume at day 42 − tumor volume at day 0)/42, confirmed that MAFG shRNA or miR-4660 overexpression robustly inhibited pOS-1 xenograft growth in SCID mice (Figure 8B). At day 42, tumors of all three groups were isolated and individually weighed. Results showed that xenografts bearing MAFG shRNA or lv-pre-miR-4660 were significantly lighter than the control xenografts (Figure 8C). Mouse body weights were not significantly different between the three groups (Figure 8D), and no significant toxicity was observed.

At day 7 and day 14, one tumor of each group was isolated, with a total six tumors obtained. Western blot assay results (Figure 8E) confirmed significant MAFG-HMOX1 downregulation in tumors bearing MAFG shRNA or lv-pre-miR-4660, where *MAFG* mRNA depletion was detected (Figure 8F). As shown, miR-4660 overexpression was detected in tumor tissues bearing lv-pre-miR-4660 (Figure 8G). miR-4660 expression was unchanged in MAFG shRNA-expressing tumor tissues (Figure 8G ). *In vivo* RNA-FISH experiments confirmed *MAFG* mRNA downregulation in pOS-1 xenografts bearing MAFG shRNA or lv-pre-miR-4660 (Figure 8H). Thus, consistent with the *in vitro* findings, MAFG silencing or ectopic miR-4660 overexpression inhibited subcutaneous OS xenograft growth in SCID mice.

To further support a role of the miR-4660-MAFG axis in OS cell growth *in vivo*, genetically modified primary OS cells were injected into the proximal tibia of nude mice to establish an animal model of *in situ* OS. These cells included stable pOS-1 cells expressing MAFG shRNA lentivirus (MAFG shRNA), pre-miR-4660 lentivirus (lv-pre-miR-4660), or the control virus (miRC+shC). Results demonstrated that, 24 days (day 24) after the cell injection, the volumes of *in situ* pOS-1 xenografts bearing MAFG shRNA or lv-pre-miR-4660 were significantly lower than xenografts with control virus (Figures 8H and 8I). At day 24, the *in situ* pOS-1 xenografts were isolated and homogenized. As demonstrated, *MAFG* mRNA levels were significantly decreased in *in situ* pOS-1 xenograft tissues with MAFG shRNA and lv-pre-miR-4660 (Figure 8J). The mature miR-4660 levels were increased over eight-fold in lv-pre-miR-4660-expressing xenograft tissue (Figure 8K). These results demonstrate that MAFG shRNA or forced miR-4660 overexpression potently inhibits OS xenografts *in situ* growth in mice.

## Discussion

MAFG forms a heterodimer with Nrf2 to bind ARE, initiating transcription of detoxification enzymes and antioxidant genes.^35^, ^36^, ^37^ Recent studies suggest that MAFG could play an important role in cancer progression.^38^ The overexpression of MAFG in hepatocellular carcinoma is associated with tumor progression and decreased survival.^38^ In non-small cell lung cancer, MAFG inhibition or downregulation induced ROS production, sensitizing cancer cells to cisplatin-induced apoptosis.^39^ BRAF^V600E^-stabilized MAFG initiated recruitment of a co-repressor complex to CpG island methylator phenotype (CIMP) gene promoters in colorectal cancer cells, which is associated with tumorigenesis and cancer growth.^40^ Silencing of MAFG potently inhibited colorectal cancer cell growth.^40^

We provide evidence to show that MAFG is a novel oncogenic gene and potential therapeutic target of OS. *MAFG* mRNA and protein expression are significantly elevated in human OS tissues, compared to low expression in normal bone tissues and osteoblasts. In primary and established OS cells, MAGF shRNA or KO inhibited OS cell growth, proliferation, and migration, while simultaneously eliciting cell-cycle arrest and cell apoptosis. Conversely, ectopic overexpression of MAFG further promoted OS cell progression *in vitro*. Significantly, MAFG silencing potently inhibited growth of subcutaneous OS xenografts and *in situ* OS xenografts in mice.

Bai et al.^41^ have reported that low levels of ROS, detected in human OS tissues and cells, are important for OS cell proliferation and invasion. However, sustained ROS production and profound oxidative injury induced OS cell growth arrest and apoptosis. Wang et al.^42^ have shown that andrographolide induced OS cell apoptosis via ROS production and downstream JNK activation. Similarly, hyperthermia-induced OS cell apoptosis was associated with ROS production.^43^ Ionizing radiation (IR) was also found to induce robust ROS production to promote U2OS cell apoptosis.^44^ Such action by IR was attenuated by Nrf2 upregulation-induced antioxidant response.^44^ We found that MAFG silencing results in Nrf2-signaling inhibition, leading to significant ROS production and oxidative injury. Antioxidants, including NAD, PDTC, and AGI-1067, inhibited MAFG silencing-induced OS cell apoptosis.

miRNA binds the 3′ UTR of the complementary mRNAs, causing targeted mRNA translation inhibition and/or mRNA degradation.^25^^,^^26^ miRNA is often dysregulation in human OS^22^, ^23^, ^24^ and is associated with tumorigenesis progression.^25^^,^^26^ The potential function of miR-4660 is largely unknown. A recent study by Tu et al.^45^ has demonstrated that miR-4660 is downregulated in patients with oxalosis. It bound directly to 3′ UTR of alanine–glyoxylate aminotransferase (AGXT) to inhibit its expression.^45^ The results of this study demonstrate that miR-4660 is a novel MAGF-targeting miRNA in OS cells. miR-4660 is localized in the cytoplasm of OS cells, where it can bind directly to 3′ UTR of *MAFG* mRNA. In OS cells, ectopic overexpression of miR-4660 inhibited MAFG 3′ UTR luciferase activity and expression. Both were, however, augmented with miR-4660 inhibition. miR-4660 overexpression mimicked MAFG silencing-induced actions, inhibiting OS cell proliferation and migration and inducing ROS production and apoptosis activation. Importantly, restoring MAFG expression completely reversed miR-4660 overexpression-induced OS cell inhibition. Furthermore, exogenously altering miR-4660 expression failed to affect the function of MAFG-KO OS cells. Significantly, decreased miR-4660 expression in human OS tissues correlates with MAFG upregulation.

OS accounts for 60% of the common histological subtypes of bone sarcoma. It is the primary malignant bone tumor that commonly affects adolescents and young adults.^6^^,^^46^ Current chemotherapy for human OS consists of the combination of methotrexate, doxorubicin, and cisplatin (MAP).^2^^,^^6^^,^^47^ The application of molecularly targeted therapies has so far failed to significantly improve the overall survival of advanced OS patients. Hence, further exploration of the pathology of OS is essential for improving the prognosis of patients with advanced OS.^2^^,^^6^^,^^47^ Our results indicate that dysregulation of the miR-4660-MAFG axis is involved in the progression of OS. Targeting the miR-4660-MAFG axis could be a promising therapeutic strategy for this devastating malignancy.

## Materials and methods

### Chemicals and reagents

CCK-8 was obtained from Dojindo (Kumamoto, Japan). Antioxidants including NAC, PDTC, and AGI-1067, as well as puromycin, neomycin, polybrene, and Matrigel were provided by Sigma-Aldrich Chemicals (St. Louis, MO, USA). Cell culture reagents, including fetal bovine serum (FBS), DMEM, and antibiotics, were obtained from Hyclone (Logan, UT, USA). Antibodies of MAFG, HMOX1, and tubulin were provided by Cell Signaling Technology (Beverly, MA, USA).

### Cell culture

Established human OS cell lines, U2OS and MG63, were obtained from the Cell Bank of Shanghai Institute of Biological Science (Shanghai, China). Cells were cultured using a previously described protocol.^48^ The primary human OS cells derived from three human OS patients, pOS-1, pOS-2, and pOS-3, were provided by Dr. Ji at Nanjing Medical University,^49^ and cells were cultured under the described conditions.^49^^,^^50^ Primary OS cells at passages 3–10 were utilized. OB-6 human osteoblastic cells and primary human osteoblasts were provided again by Dr. Ji at Nanjing Medical University, cultured as previously described.^51^^,^^52^ The protocols of the study were approved by the institutional animal care and use committee (IACUC) and ethics committee of Soochow University.

### Human OS tissues

Human OS tissues and the matched surrounding normal bone tissues were from a set of 12 OS patients with written informed consent administrated at the Affiliated Children Hospital of Soochow University (Suzhou, China). Tissues were incubated with the tissue lysis buffer^53^ and stored in liquid nitrogen. The protocols of using human tissues were approved by the ethics committee of Soochow University.

### Quantitative real-time PCR

TRIzol reagents were applied to achieve total RNA, which was quantified and reversely transcribed.^54^ The quantitative real-time PCR was performed by the SYBR Premix Ex TaqTM kit under the ABI-7500 PCR system (Shanghai, China).^54^ mRNA expression was quantified by 2^−ΔΔCt^ protocol with glyceraldehyde-3-phosphatedehydrogenase (GAPDH) as the internal control. Mature miR-4660 expression was detected using the TaqMan microRNA assay of hsa-miR-4660 (Applied Biosystems, Shanghai, China). The TaqMan MicroRNA Reverse Transcription kit (Applied Biosystems) was used for RNA (10 ng) reverse transcription via the stem-loop primer. *U6 RNA* was tested as the internal control for miR-4660. mRNA primers were listed in Table 1. mRNA primers of human *GCLC*, *NQO1*, and *GAPDH* were described previously.^55^ mRNA primers for *MAFF* and *MAFK* were provided by Genechem (Shanghai, China).

### MAFG shRNA

OS cells were initially seeded into six-well plates at 1 × 10^5^ cells per well. MAFG shRNA lentiviral particles (shRNA sequences are listed in Table 1; Genechem) were added to cultured OS cells. After 24 h, puromycin-containing medium was added to the OS cells, with stable cells (sh-MAFG cells) achieved within 4–5 passages. MAFG silencing was verified by quantitative real-time PCR and western blotting assays.

### MAFG KO

Human OS cells were initially seeded into six-well plates at 1 × 10^5^ cells per well. The MAFG small guide RNA (sgRNA, Target DNA sequence: *TGAGAATGGCACCAGCCTGA*, PAM Sequence: *CGG*) was inserted into the CRISPR-Cas9 PX458 construct.^56^ The construct was transfected to human OS cells by Lipofectamine 2000 (Thermo Fisher Invitrogen, Shanghai, China). The transfected cells were inoculated into 96-well plates to establish single monoclonal cells, subjected to screening of MAFG KO. The established cells were further subjected to selection by puromycin (5.0 μg/mL)-containing medium, and stable cells were established (ko-MAFG). MAFG KO was verified by quantitative real-time PCR and western blotting assays.

### Forced MAFG overexpression

The full-length MAFG cDNA and the UTR null MAFG cDNA were synthesized and sequence-verified by Genechem (Shanghai, China) and separately inserted into the GV369 construct (Genechem). The construct and the lentivirus-packing plasmids (psPAX2 and pMD2.G, from Dr. Jiang^57^) were co-transfected to the packaging cell line (HEK293T), generating MAFG-expressing lentivirus. The viruses were then enriched, filtered, and added to OS cells (cultured in complete medium with polybrene) for 24 h. Thereafter, puromycin (5.0 μg/mL)-containing complete medium was added to select stable cell line OE-MAFG, where MAFG overexpression was verified by quantitative real-time PCR and western blotting assays. Control cells were transduced with the empty vector.

### Forced overexpression or inhibition of miR-4660

The miR-4660 precursor (pre-miR-4660) sequence (ACUCCUUCUGCAGCUCUGGUGGAAAAUGGAGAAGACUUUUCCUUUCCUCCAUCUCCCCCAGGGCCUGGUGGAGU) and the corresponding anti-sense sequence were synthesized and verified by Genechem. Each was individually inserted into a GV248 lentiviral construct (Genechem, Shanghai, China). The construct and the lentivirus-packing plasmids (psPAX2 and pMD2.G) were co-transfected into HEK293T cells, establishing pre-miR-4660 expression lentivirus (lv-pre-miR-4660) or pre-miR-4660 anti-sense lentivirus (lv-antagomiR-4660). Viruses were added to cultured OS cells. To select stable cell lines, puromycin (5.0 μg/mL) was added. The mature miR-4660 (sequence: UGCAGCUCUGGUGGAAAAUGGAG) expression was examined by quantitative real-time PCR in stable cells. Control OS cells were transduced with lentiviral nonsense control miRNA construct (lv-miRC^58^) or the lentiviral nonsense control miRNA inhibitor (anta-C^58^).

### Transfection of miR-4660 mimics

OS cells were initially seeded into six-well plates at 1 × 10^5^ cells per well. Cells were transfected with 200 nM of WT miR-4660 (UGCAGCUCUGGUGGAAAAUGGAG) or the mutant one (UGC*U*GCUCUGGUGGAAAAUGGAG) using Lipofectamine 2000 (Invitrogen). After 24 h, the transfection was repeated for one more round. The control cells were transfected with scramble nonsense control miRNA (miRC).

### MAFG 3ʹ UTR luciferase reporter assay

As previously described,^59^ the MAFG 3ʹ UTR sequence containing predicted miR-4660 binding sites (at positions 788–795, 5′-AGAGCUGCA-3′) was amplified by Genechem and sub-cloned into a pMIR-REPORT miRNA expression reporter vector (provided by Dr. Chen at Jiangsu University^60^), generating the luciferase reporter plasmid pMIR-MAFG-UTR. The plasmid was transfected to the cultured OS cells by Lipofectamine 2000. Cells were then subjected to the applied genetic treatments, with luciferase activity examined by a dual-luciferase reporter assay system (Promega, Suzhou, China).

### RNA pull-down

A total of 8 × 10^6^ OS cells, transfected with a biotinylated-miR-4660 mimic (WT or Mut) or control mimic (100 nM each), were trypsinized and incubated with the cell lysis buffer. RNA-IP was carried out by adding the streptavidin-coated magnetic beads^61^ into the cell lysates. The bound mRNA was purified utilizing the RNeasy mini kit (QIAGEN, Shanghai, China). The captured *MAFG* mRNA expression was tested by quantitative real-time PCR and normalized to the input controls.

### RNA-FISH

RNA-FISH assay was carried out via a FISH kit (RiboBio, Guangzhou, China). Briefly, fluorescein isothiocyanate (FITC)-labeled *MAFG* mRNA probe (green) and Cy3-labeled miR-4660 probe (red) (RiboBio) were added to pOS-1 cells (at 37°C for 12 h). Cells were then rinsed and observed under a fluorescence microscope. FITC-labeled *MAFG* mRNA probe was also added to xenograft tumor slides and visualized under a fluorescence microscope.

### RNA-IP

Cells were first incubated with the complete RIP lysis buffer (Beyotime). For each treatment, 1,000 μg total cell lysates were incubated with magnetic beads conjugated with anti-Argonaute 2 (Ago2, Santa Cruz Biotechnology) antibody or an anti-IgG antibody (Santa Cruz Biotechnology) overnight. Beads were washed and incubated with Proteinase K (Sigma). Finally, quantitative real-time PCR was performed to test the purified RNAs; *MAFG* mRNA and miR-4660 levels were normalized to input controls.

### Western blotting

Western blotting assays were performed by well-established protocols as in our previous studies.^48^^,^^62^ The same set of lysates was run separately in sister gels when testing different proteins. The band intensity was quantized by densitometric analysis using NIH ImageJ software.

### Cell viability

Cell viability was assessed through a CCK-8 assay. Briefly, cells with the applied genetic modifications were seeded into the 96-well tissue culture plates (at 3 × 10^3^ cells per well). Following incubation for 96 h, cell viability was estimated by recording the CCK-8 OD at 450 nm using a microplate reader.

### EdU staining

OS cells with the applied genetic modifications were seeded into six-well plates (at 1 × 10^5^ cells in each well) and cultured for 72 h. An EdU Apollo-567 assay kit (RiboBio, Guangzhou, China) was employed to quantitatively measure cell proliferation. The cell nuclei were stained with both EdU and DAPI, visualized under a fluorescent microscope (Leica, DM 4000, Germany). Cells in each field of view were then counted and analyzed. The nuclear EdU ratio, % versus DAPI, from at least 1,000 cells in five random views per treatment was calculated.

### *In vitro* cell migration assay

On the upper Transwell chambers (8-μm pore, Corning, New York, NY, USA) OS cells (10,000 cells per chamber, in serum-free medium) with applied genetic modifications were plated,^63^ with the lower chambers filled with 10% FBS complete medium. After 16 h, OS cells migrating to the lower chambers were fixed, stained, and counted.

### Apoptosis detection

OS cells with the applied genetic modifications were seeded into six-well plates (at 1 × 10^5^ cells in each well) and cultured. The detailed protocols for cell apoptosis assays, including nuclear TUNEL staining, Annexin V FACS, and caspase-3 activity assay, were described in the previous studies.^64^^,^^65^

### ROS detection

OS cells with applied genetic treatments were seeded into the six-well plates at 1 × 10^5^ cells per well and stained with CellROX fluorescence dye (Thermo Fisher Scientific, Shanghai, China) for 1 h in the dark. The CellROX fluorescence intensity (at 625 nm) was detected and the representative CellROX images were presented as well.

### JC-1 assay

OS cells were seeded into the six-well plates and stained with JC-1 fluorescence dye for 30 min. JC-1 intensity was quantified via a fluorescence spectrofluorometer (F-7000, Hitachi, Japan) at test-wavelength of 488 nm (green). The representative JC-1 images, integrating both green fluorescence (at 488 nm) and red fluorescence (at 625 nm) channels, were presented as well.

### ARE reporter assay

The primary human OS cells, pOS-1, were initially seeded in six-well plates at 50%–60% confluence. Cells were then transfected with the ARE-inducible firefly luciferase vector (from Dr. Jiang at Nanjing Medical University^57^). Following the applied genetic treatment, total cell lysates (30 μg per treatment) were subjected to ARE-reporter luciferase activity assay under a luminescence machine.

### Mice xenograft assay

The CB.17 female SCID mice and the nude mice were maintained under the Animal Facility of Soochow University (Suzhou, China). For each mouse, 5 × 10^6^ OS cells (in Matrigel-containing medium, no serum) were s.c. injected to the flanks. The subcutaneous OS xenografts were established within 3 weeks with each tumor close to 100 mm^3^ in volume. Tumor-bearing mice were randomly divided into three groups and were treated as described. Mice body weight and bidimensional tumor measurements were recorded every 7 days. Tumor volume was estimated using the standard formula: (length × width2)/2. For the *in situ* OS model, pOS-1 cells (3 × 10^6^ cells per mouse) with applied genetic modifications were injected to the proximal tibia of the nude mice. Twenty-four days after cell injection, *in situ* tumors were visualized under X-ray film. Tumors were isolated and measured and tumor tissues tested by quantitative real-time PCR analyses. The animal protocols were approved by IACUC and Ethic Committee of Soochow University.

### Statistical analysis

Statistical analysis was performed with SPSS version 23.0 (SPSS, Chicago, IL, USA). All quantitative data were presented as the mean ± standard deviation (SD). Differences between two groups were compared by the Student’s t test when they had a normal distribution. A one-way analysis of variance (ANOVA) followed by a Scheffé and Tukey test was used to compare data among groups when they had a normal distribution and homogeneous variances. p values less than 0.05 were considered statistically significant.

## Supplemental information

Document S1. Figures S1–S3

Document S2. Article plus supplemental information
