# Supplementary material for: MAFG-driven osteosarcoma cell progression is inhibited by a novel miRNA miR-4660
Source: Mol Ther Nucleic Acids. 2021 Mar 13;24:385–402. doi: 10.1016/j.omtn.2021.03.006 (PMC8039776; doi:10.1016/j.omtn.2021.03.006)
Supplement: Document S1. Figures S1–S3 [file mmc1.pdf]

OMTN, Volume *24*

Supplemental information

MAFG-driven osteosarcoma cell progression is inhibited by a novel miRNA miR-4660

Hua-jian Shan, Lun-qing Zhu, Chen Yao, Zhi-qing Zhang, Yuan-yuan Liu, Qin Jiang, Xiao-zhong Zhou, Xiao-dong Wang, and Cong Cao

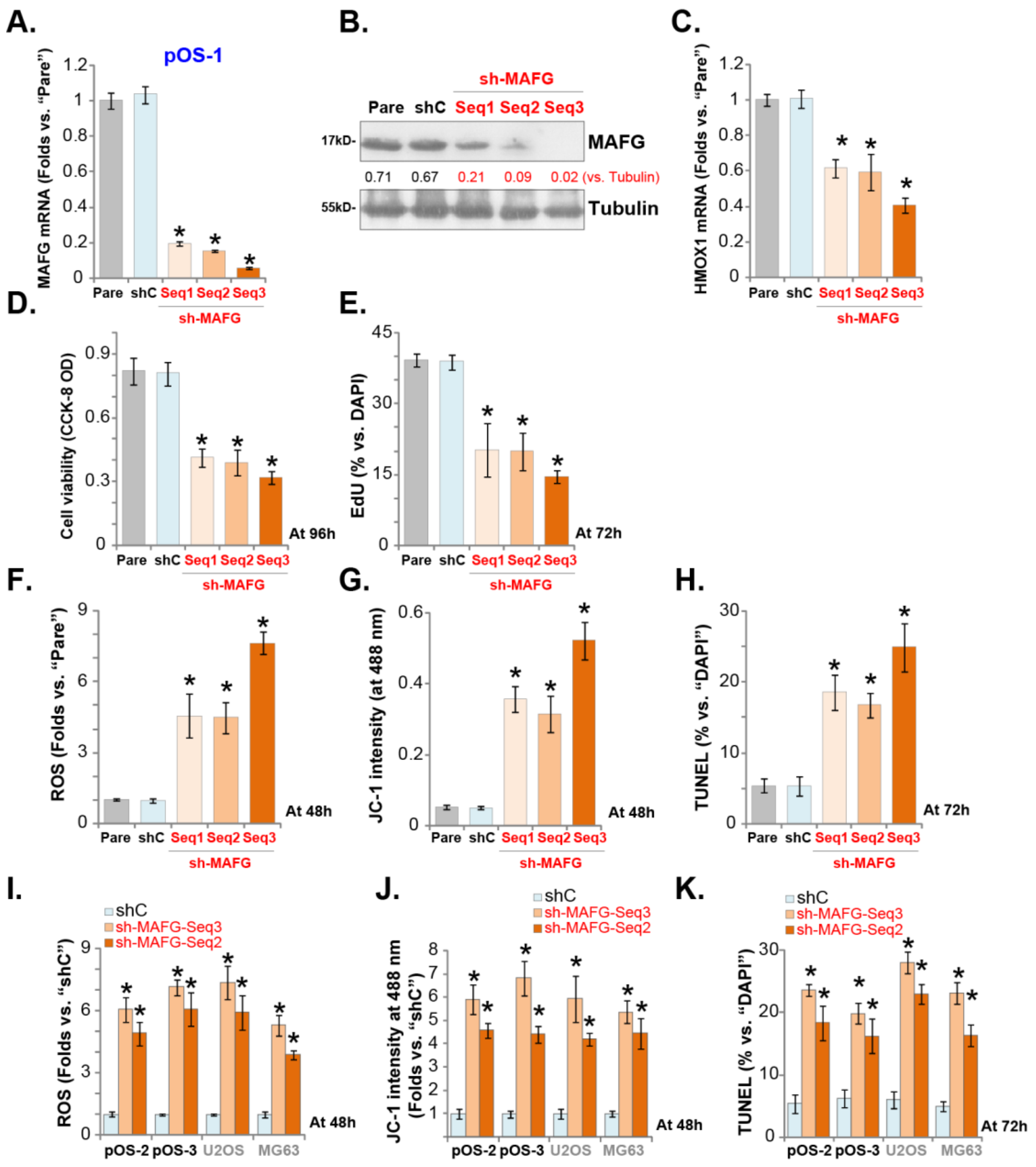

Figure S1. pOS-1 cells, bearing applied MAFG shRNA ("sh-MAFG-Seq1/2/3", with non-overlapping sequences) or scramble control shRNA (shC) were established, and expression of listed genes tested qPCR and Western blotting assays (**A**-**C**); Cells were cultured for applied time periods, cell viability (CCK-8 OD, **D**), proliferation (by recording nuclear EdU ratio, **E**), as well as ROS contents (CellROX intensity, **F**), mitochondrial depolarization (JC-1 green monomers intensity, **G**) and apoptosis intensity (nuclear TUNEL ratio, **H**) were tested by the assays mentioned in the text, with results quantified. Stable primary human OS cells (pOS-2/pOS-3, derived from OS patients) or the established OS cell lines (U2OS and MG63), bearing MAFG shRNA (sh-MAFG-Seq3/Seq2) or scramble control shRNA (shC), were cultured for applied time periods, ROS contents (**I**), mitochondrial depolarization (**J**) and cell

apoptosis (**K**) were tested similarly, with results quantified. Data were presented as mean ± standard deviation (SD, n=5). * $P < 0.05$ vs. "shC" cells. Experiments in this figure were repeated five times with similar results obtained.

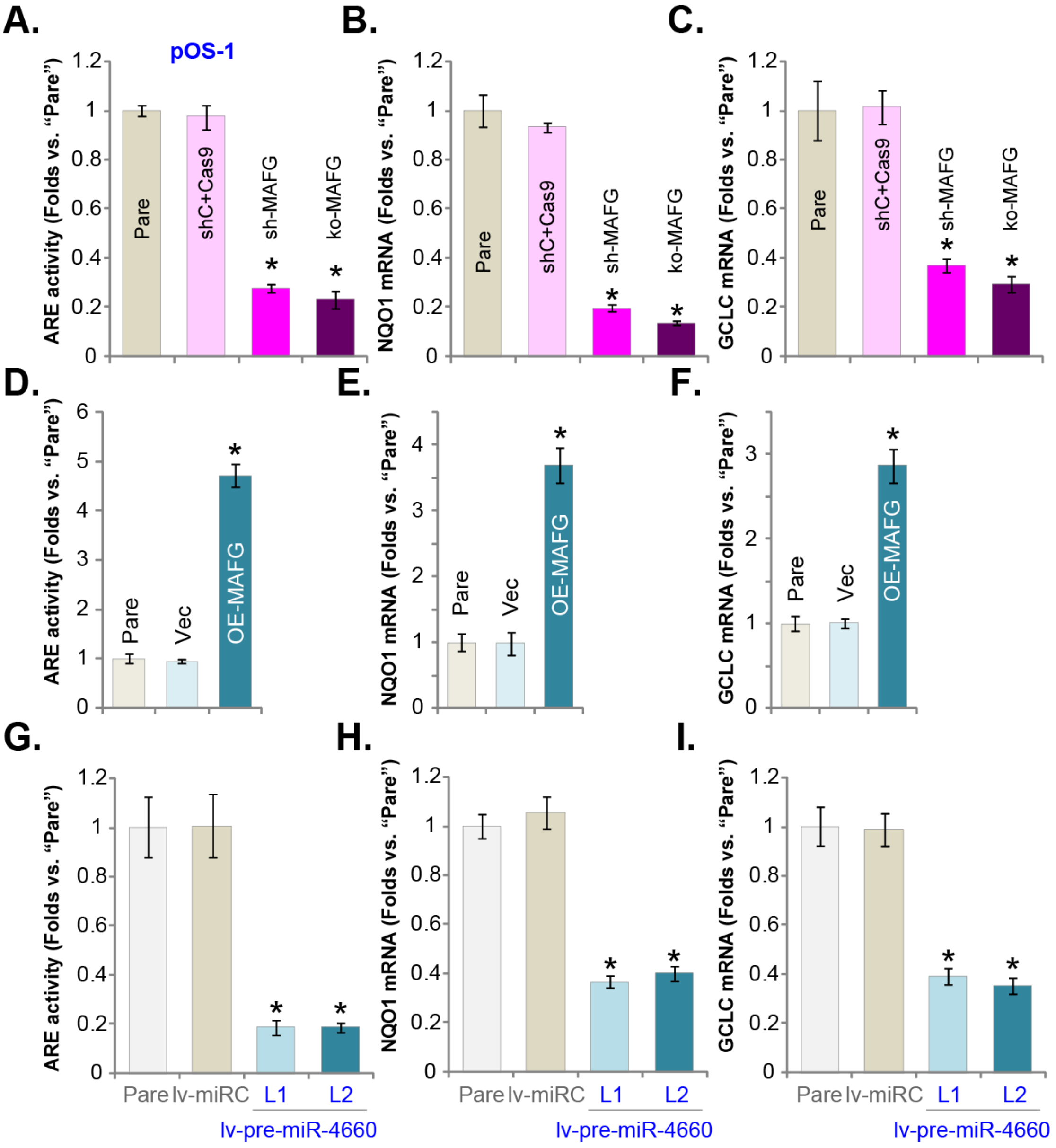

Figure S2. Stable pOS-1 cells bearing MAFG shRNA (sh-MAFG), the lenti-CRISPR/Cas9-MAFG-KO construct (ko-MAFG), scramble control shRNA plus CRISPR/Cas9 empty vector (shC+Cas9), as well as MAFG-expressing GV369 vector ("OE-MAFG"), the empty vector ("Vec"), or the lentiviral pre-microRNA-4660 ("lv-pre-miR-4660", two lines) were established, the relative ARE-reporter luciferase activity was measured (**A**, **D** and **G**); Expression of listed mRNAs was tested by qPCR assays (**B**, **C**, **E**,

F, H and I). Data were presented as mean ± standard deviation (SD, n=5). * $P < 0.05$ vs. "Pare" cells. Experiments in this figure were repeated five times with similar results obtained.

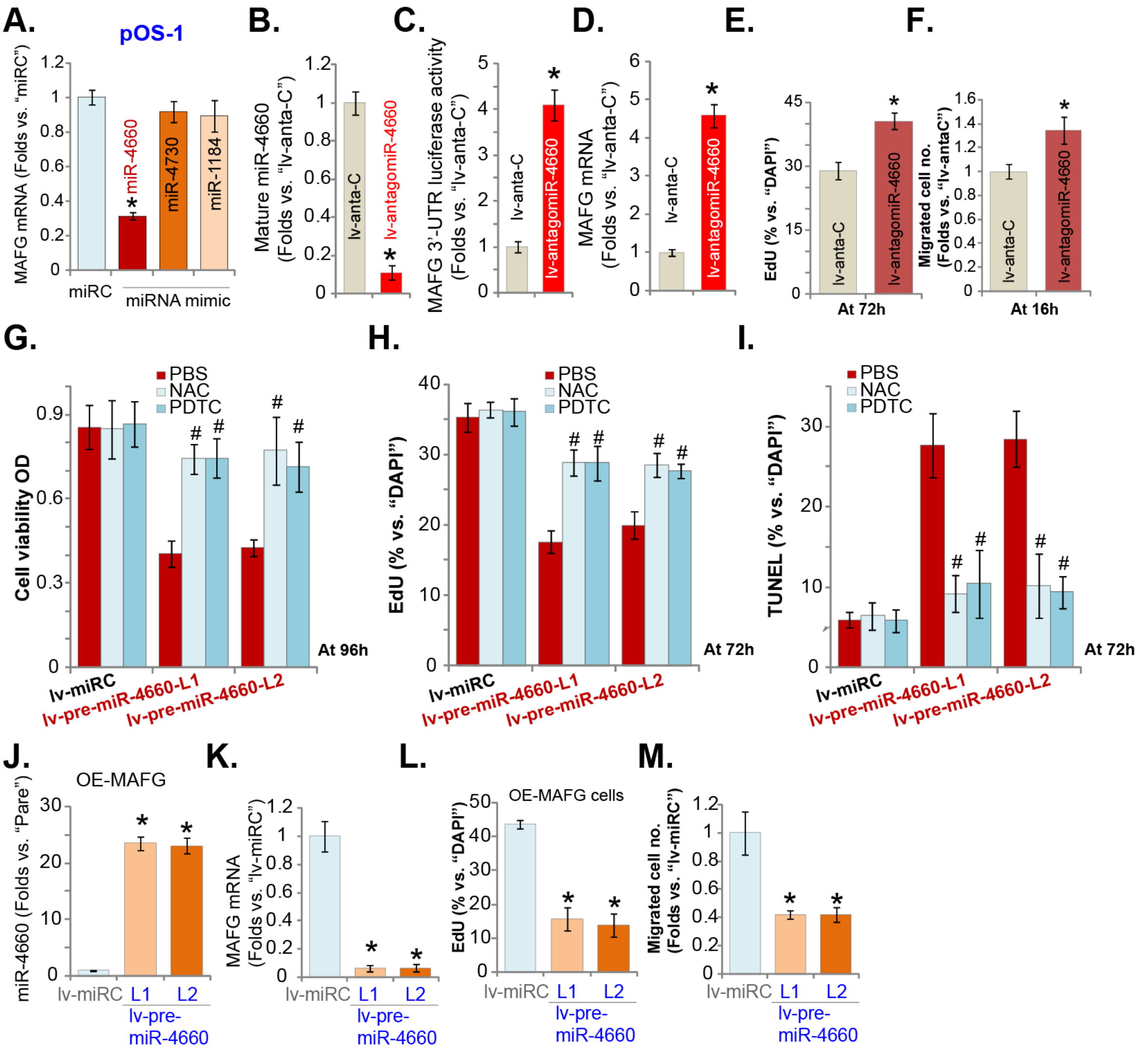

Figure S3. pOS-1 cells were transfected with 500 nM of non-sense microRNA control ("miRC"), or the applied miRNA mimic (500 nM) for 48h, expression of *MAFG* mRNA was tested by qPCR (**A**). Stable pOS-1 cells, with the pre-miR-4660 anti-sense construct (lv-antagomiR-4660) or the control anti-sense construct (lv-anta-C), were established; Expression of listed genes (**B** and **D**) and MAFG 3'-UTR luciferase reporter activity (**C**) were tested. Cells were cultured for applied time periods, cell proliferation (by recording nuclear EdU incorporation, **E**) and migration (**F**) were tested, with results quantified Stable pOS-1 cells expressing the lentiviral pre-microRNA-4660 ("lv-pre-miR-4660-L1" and "lv-pre-miR-4660-L2") were treated with NAC (50 μM), PDTC (40 μM) or PBS, cells were then cultured for applied time periods; Control cells with lentiviral non-sense microRNA ("lv-miRC") were left untreated; Cell viability, proliferation

and apoptosis were tested by CCK-8 (**G**), nuclear EdU staining (**H**) and TUNEL staining (**I**) assays, respectively, and results were quantified. MAFG overexpressed pOS-1 cells, "OE-MAFG", were transduced with lentiviral pre-microRNA-4660 ("lv-pre-miR-4660"). With selection by puromycin two stable cell lines were established; Control cells were transfected with lentiviral non-sense microRNA ("lv-miRC"). Expression of miR-4660 and MAFG mRNA was tested by qPCR assays (**J** and **K**); Cell proliferation (**L**) and migration (**M**) were tested similarly and results were quantified. Data were presented as mean ± standard deviation (SD, n=5). * $P < 0.05$ vs. "miRC" (**A**). $^{\#}$ $P < 0.05$ vs. PBS group (**G**-**I**). * $P < 0.05$ vs. "lv-anta-C" cells (**B**-**F**). * $P < 0.05$ vs. "lv-miRC" cells (**J**-**M**). Experiments in this figure were repeated five times with similar results obtained.
